# Synthesis and Characterization of ZnO-Nanostructured Particles Produced by Solar Ablation

**DOI:** 10.3390/ma16196417

**Published:** 2023-09-26

**Authors:** Adriana-Gabriela Schiopu, Mihai Oproescu, Vasile Gabriel Iana, Catalin Marian Ducu, Sorin Georgian Moga, Denisa Stefania Vîlcoci, Georgiana Cîrstea, Valentin Marian Calinescu, Omar Ahmed

**Affiliations:** 1 Faculty of Mechanics and Technology, National University of Science and Technology POLITEHNICA Bucharest—Pitești University Centre, Targu din Vale, No. 1, 110040 Pitesti, Romania; catalin.ducu@upit.ro; 2 Faculty of Electronics, Communication and Computers, National University of Science and Technology POLITEHNICA Bucharest—Pitești University Centre, Targu din Vale, No. 1, 110040 Pitesti, Romania; gabriel.iana@upit.ro; 3 Regional Center of Research & Development for Materials, Processes and Innovative Products Dedicated to the Automotive Industry (CRCD-AUTO), National University of Science and Technology POLITEHNICA Bucharest—Pitești University Centre, Targu din Vale, No. 1, 110040 Pitesti, Romania; sorin.moga@upit.ro (S.G.M.); georgiana.cirstea@upit.ro (G.C.); 4 Interdisciplinary Doctoral School, National University of Science and Technology POLITEHNICA Bucharest—Pitești University Centre, Targu din Vale, No. 1, 110040 Pitesti, Romania; valentin.calinescu@upit.ro (V.M.C.); omaralssadi@yahoo.com (O.A.)

**Keywords:** nanoparticles, solar energy, ablation, metal oxides

## Abstract

Nowadays, nanotechnology offers opportunities to create new features and functions of emerging materials. Correlation studies of nanostructured materials’ development processes with morphology, structure, and properties represent one of the most important topics today due to potential applications in all fields: chemistry, mechanics, electronics, optics, medicine, food, or defense. Our research was motivated by the fact that in the nanometric domain, the crystalline structure and morphology are determined by the elaboration mechanism. The objective of this paper is to provide an introduction to the fundamentals of nanotechnology and nanopowder production using the sun’s energy. Solar energy, as part of renewable energy sources, is one of the sources that remain to be exploited in the future. The basic principle involved in the production of nanopowders consists of the use of a solar energy reactor concentrated on sintered targets made of commercial micropowders. As part of our study, for the first time, we report the solar ablation synthesis and characterization of Ni-doped ZnO performed in the CNRS-PROMES laboratory, UPR 8521, a member of the CNRS (French National Centre for Scientific Research). Also, we study the effect of the elaboration method on structural and morphological characteristics of pure and doped ZnO nanoparticles determined by XRD, SEM, and UV-Vis.

## 1. Introduction

Nanoparticles are nano-objects with all external dimensions in the nanoscale, according to the International Organization for Standardization (ISO), where the lengths of the longest and shortest axes of nano-objects do not differ significantly (ISO/TS 80004-2:2015) [1]. The synthesis of nanoparticles is possible using a variety of methods, which can be broadly classified into two categories: top-down and bottom-up.

Top-down methods involve physically breaking down a bulk material into smaller particles. This can be done using techniques such as milling [2], laser ablation [3], and sputtering [4]. Top-down methods are typically used to synthesize nanoparticles with a narrow size distribution, but they can be time-consuming and expensive.

Bottom-up methods involve building up nanoparticles from smaller molecules or atoms. This can be done using techniques such as chemical reduction [5], sol-gel synthesis [6], hydrothermal synthesis [7], and microemulsion [8]. Bottom-up methods are typically more versatile than top-down methods and can be used to synthesize a wider variety of nanoparticles.

A comparison of the most used nanoparticle synthesis methods can be found in Table 1.

Going forward, an innovative process to prepare nanoparticles is explained: solar ablation (SA).

Solar ablation is the process of removing material from a surface using the heat of sunlight. The process is similar to laser ablation, electron beam ablation, or plasma ablation but also has certain peculiarities. The solar wavelength is in the range of 100 nm–1 mm, while the laser wavelength is typically in the range of 100–10,000 nm. Solar ablation has the advantage that it does not use chemicals or solvents and is environmentally friendly, only that the solar energy depends on geographical coordinates and atmospheric conditions that are typically in the range of 1–1000 watts/m^2^.

Solar ablation (SA), also called solar physical vapor deposition (SPVD), was used to prepare ZnO, Zn1-xAlxO, Zn1-xCoxO, Zn1-xBixO, metallic Zn nanophases, TiO_2_ and Fe, Co, Mn-doped TiO_2_ [9] or ZrO_2_ [10] in “heliotron” type solar reactors in 2 kW furnaces. Also, in the same solar furnace were synthesized carbon nanoparticles obtained starting from Pd/C [11], long single-walled nanotubes [12] using graphite as a precursor and carbon microparticles from polymer waste radiation [13].

Solar ablation has a number of potential applications, including:

- Solar propulsion: Solar ablation can be used as a force by evaporating a material from the bulk surface.
- Solar thermal energy: Solar ablation can be used to generate heat by evaporating a material, such as water, from a solar collector. The heat can then be used to generate electricity or to heat water [14].
- Solar micromachining: Solar ablation can be used to create small, precise features on a surface, such as those used in integrated circuits.

Ablation with solar energy is a promising technology with many possibilities. However, it is still in the early stages of development, and there are a number of challenges that need to be addressed before it can be widely used.

Here are some of the challenges of solar ablation:

- Controlling the ablation process: The ablation process is very sensitive to ray power, beam spot size, and pulse duration. It is difficult to control these parameters precisely, which can lead to uneven ablation and poor surface quality.
- Minimizing material damage: The solar ablation process can also damage the underlying material, which can reduce the lifetime of the ablated surface.
- Increasing the ablation rate: The ablation rate is typically very slow, which limits the applications of solar ablation.

Despite these challenges, solar ablation is a promising technology with a wide range of potential applications. As the technology continues to develop, it is likely to become more widely used in the future.

It will be necessary to consider the specific properties of the nanoparticles when selecting the synthesis method. In some cases, it may be necessary to use a combination of methods to obtain the desired properties.

The characterization of nanoparticles is a complex and challenging task, as they exhibit a wide range of properties that can be affected by their size, shape, composition, and surface chemistry. The following are some of the most common techniques used to characterize nanoparticles: Scanning electron microscopy (SEM) or transmission electron microscopy (TEM) [15], dynamic light scattering (DLS) [16], UV-Vis spectroscopy [17], X-ray diffraction (XRD) [18], Fourier transform infrared spectroscopy (FTIR) [19], X-ray spectroscopy (EDS) or X-ray fluorescence (XRF) [20], and Zeta potential [21].

The choice of characterization technique will depend on the specific properties of the nanoparticles that are being studied. In some cases, it may be necessary to use a combination of techniques to obtain a complete characterization of the nanoparticles.

Physicochemical properties of functional nanoparticles have recently drawn considerable attention due to their advantages in various applications [22,23]. For example, nanoparticles not only can be used for optical properties [24], electric properties [25], magnetic properties [23,26,27,28], and catalytic properties [29] but also for biological properties [30,31], life science, environmental technology [32], diagnosis, and therapy [33,34,35,36].

Films or coatings, as well as massive nanomaterials, can be prepared directly from oxide nanopowders resulting from various methods from about 20 years ago: precipitation [37,38], coprecipitation [39], sol-gel [40], hydrothermal [41], microwave synthesis [42], pyrolysis [43], decomposition [44], sonoelectrodeposition [45], sonochemistry [46], impregnation [47], mechanosynthesis [48], and chemical vapor deposition [49].

From the great variety of emerging metal oxides, nano zinc oxide materials demonstrate antifungal, antibacterial, photochemical, catalytic, electrical, anticorrosive, photovoltaic, and UV filtering properties [50,51,52,53,54,55]. These properties make it useful in cosmetics, paints, coatings, sun care, and antibacterials. As a result of the material’s ability to adopt a wide variety of morphologies, it has a wide range of applications. By means of different fabrication conditions, zinc oxide can be shaped into nanostructures [56]: nanowires [57], nanotubes [58], nanorods [58], nanoribbons [59], nanoneedles [60], nanocables [61], nanowhiskers [62], and polyhedral [63,64] with innovative properties.

The paper structure includes, in the second section, the Experimental setup. The solar ablation of pure and Zn-doped nanoparticles was carried out experimentally using solar reactors with a specific design. The organization of the experiments took into account the solar flux, the vapor pressure, and the thickness of the material subjected to ablation. To describe the nanostructure and the composition of the nanopowders, the main method used was the X-ray diffraction (XRD) method. The nanostructure and detailed information on the nanoparticles present in the elaborated nanoparticles are studied too by scanning electron microscopy (SEM) and bright-field STEM (Scanning Transmission Electron Microscopy) in order to bring, in some cases, complementary information on the state of the nanoparticles. To identify more about their optical properties, UV-Vis spectroscopy was selected. Solar ablation (SA) nanopowders exhibit properties that are clearly related to their composition and nanostructure.

Section 3 is dedicated to the results obtained experimentally and the interpretation of these obtained results, and finally, Section 4 assumes the conclusions and future research directions.

## 2. Materials and Methods

### 2.1. Elaboration by Solar Ablation of Pure and Ni-Doped Zinc Oxide Nanoparticles 

**Figure 1:**
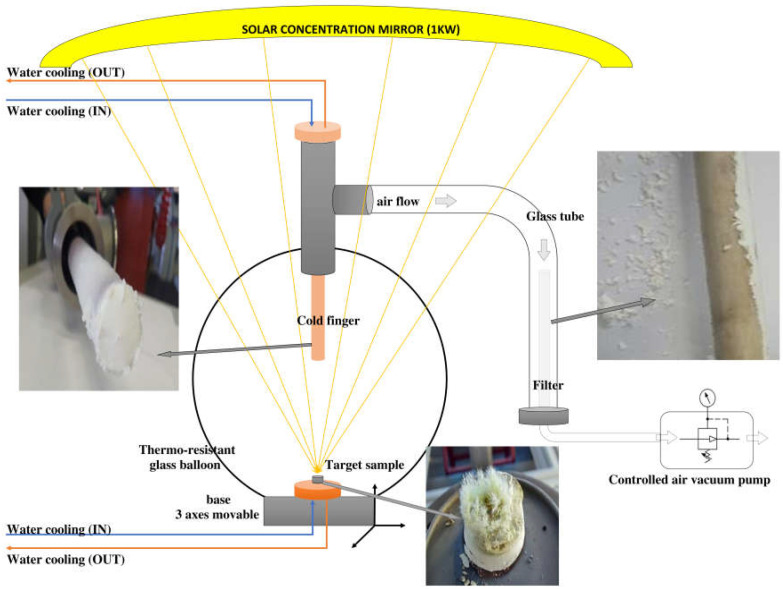
Schematic diagram of a solar reactor for ablation.

The solar radiation is concentrated in the heliotron system [65,66] in order to produce target ablation. The schematic diagram of the solar reactor during the ablation is presented in Figure 1. 

The solar ablation of nanoparticles (SANP) takes place in a reactor constituted by a thermoresistant glass balloon. The solar radiation is concentrated with the help of a parabolic mirror above the reactor. In this configuration, the collector can focus sunlight to a power of 1 kW. In the previously presented papers, the experimental collector generated two times more power [10,11,12,13]. 

The target is made by commercial sintered metal oxide powders. Targets made of mixtures of ZnO and NiO powders in mass percentages of 1:1 followed by pressing at 1tf and thermal treatment at 300 °C for 10 min were used as precursors for Ni-doping ZnO by SA. Raw materials were provided by Sigma-Aldrich and these materials were used without further purification. 

The target is placed on a copper support capable of moving in three directions (x, y, z), continuously cooled under a water circuit. Under solar radiation, the target ejects particles that then vaporize. The vapors condense on a copper support, located in the upper part of the reactor, called a cold-finger, continuously cooled too, until solid particles are formed, also on a filter connected to the glass balloon through a glass tube. The pressure around the target depends on the metal oxide and on the atmosphere inside the balloon and is controlled with a vacuum pump.

After it is subjected to concentrated solar radiation, the target has the appearance of melted ceramics, with particles on the edges in the form of sharp crystals.

The conditions for performing SA of pure and Ni-doped zinc oxide nanoparticles involved variable solar flux from 953 to 980 W/m^2^, concentrated on sintered targets under controlled air pressure (60, 100, 180 mbar) are offered in Table 2. The solar flux depends on the sunshine conditions, which can vary throughout the day. The collected amount of nanoparticles depends not only on the solar energy but also on the solar flow. For a high solar flux, quantities greater than 0.2 g are obtained, and for a pressure greater than 100 mbar, the quantity increases over 1 g.

### 2.2. Characterization of Nanoparticles

The structural characterization was determined with a Rigaku Ultima IV diffractometer, in Bragg–Brentano geometry, using CuKα radiation (45 kV, λ = 0.154 A° & 40 mA) and a D/teX Ultra one-dimensional detector with graphite monochromator. XRD patterns were purchased for phase analysis of the nanoparticles in the 2θ range of [25–101°] at a step of 0.05° and a scan speed of 2°/min. The ICDD PDF4+ 2022 database was used for crystalline phase identification [67]. In order to understand the role of dopant on the structure, the XRD data were analyzed using WPPF—whole-powder-pattern fitting—using Pdxl2. 

The morphologies were analyzed with a Hitachi 8230 Electron Microscope. The particle size by image analysis was calculated with Image J software v1.44.

Ocean Optics HR2000+ was used to measure and record the UV–Vis absorption spectra of nanoparticle solutions.

## 3. Discussion

### 3.1. Structural Analysis

**Figure 2:**
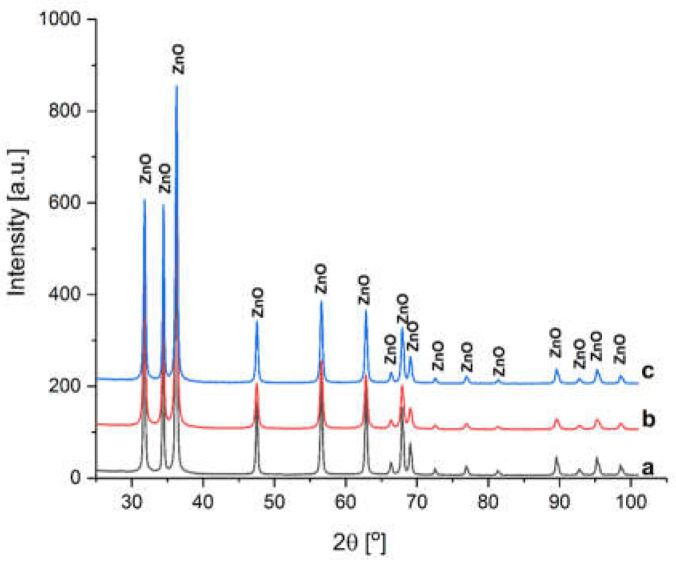
XRD patterns of the ZnO nanoparticles corresponding to (**a**) SA at 180 mbar, (**b**) SA at 60 mbar, (**c**) SA at 100 mbar.

After recording the XRD spectra for each sample of pure ZnO sample obtained after solar ablation (SANP/ZnO), all the observed diffraction peaks were indexed to a wurtzite structure in a hexagonal close-pack symmetry, space group P63mc, according to ICDD (PDF-4+ 2022) 04-016-6648 [67] as seen from Figure 2.

The perfect wurtzite structure has four-fold coordination with a hexagonal unit cell having the fraction c/a = 8 /3 = 1.633. To understand the structural properties after solar ablation, the average crystallite size D (nm) of the nanoparticles was determined using Scherrer’s relation. The lattice parameters *a* and *c* (Å), cell volume V (Å^3^), and unit cell, volume u (Å) are also presented in Table 3.

The ideal wurtzite structure is characterized by cell parameters: a = b = 3.245 Å and c = 5.199 Å; meanwhile, the cell parameters of SANP/ZnO change during the vaporization and condensation process, being influenced by the vapor pressure in the reactor. Thus, at 60 mbar, the cell parameter a changed slightly, increasing to 3.2519 Å, while the cell parameter c increased to 5.21 nm. The same evolution is observed at 180 mbar. The greatest dilatation of cell parameters is observed at a pressure of 100 mbar, such as a = 3.2531 Å and c = 5.212 Å.

The actual lattice of SANP/ZnO deviates from the ideal lattice, having a c/a ratio varying from 1.6021 to 1.6024. Also, the cell volume presented an ascending trend with a linear behavior corresponding to a pressure increase between 60 and 100 mbar. A greater pressure (180 mbar) reduces the volume of the cell but raises the crystallite size. 

**Figure 3:**
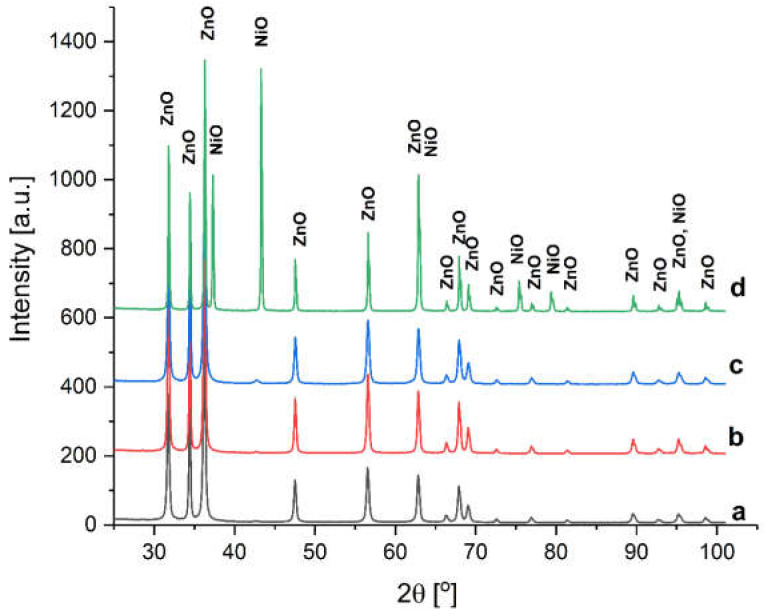
XRD patterns of the Ni-doped ZnO corresponding to (**a**) SA at 100 mbar, (**b**) SA at 180 mbar, (**c**) SA at 60 mbar, and (**d**) raw powder.

From Figure 3, the XRD patterns of Ni-doped ZnO powders demonstrate that the raw powder of ZnO doped with Ni and SANP/NiZnO corresponding to 60 mbar contains a secondary phase.

The phase peaks correspond to NiO, hexagonal structure, space group R-3 m, according to ICDD (PDF-4+ 2022) 01-078-4370 [67]. In the other Ni-doped SANP samples (SANP/NiZnO/100, SANP/NiZnO/180), no secondary phase is observed, suggesting that the Ni element may be doped into ZnO. Such a transformation is certainly to be expected if Ni ions replace Zn ions in the lattice, knowing that the Ni^2+^ ion has an ionic radius of 70 pm, while Zn^2+^ has an ionic radius of around 74 pm. Therefore, the insertion of nickel into the wurtzite structure does not promote important crystalline deformations demonstrated by the fact that the lattice of SANP of Ni-doped ZnO slightly deviates from the ideal lattice.

The c/a ratio changes from 1.6012 to 1.6014, as presented in Table 4. Also, the cell volume increases linearly with vapor pressure in the reactor. 

Comparing the specific crystallite size of the doped powders with the pure ones, a larger size can be observed in the case of the doped ones. Additional results in the literature confirm the same evolution or a smooth increase of crystallite size with Ni doping [68].

### 3.2. Morphological Analysis

**Figure 4:**
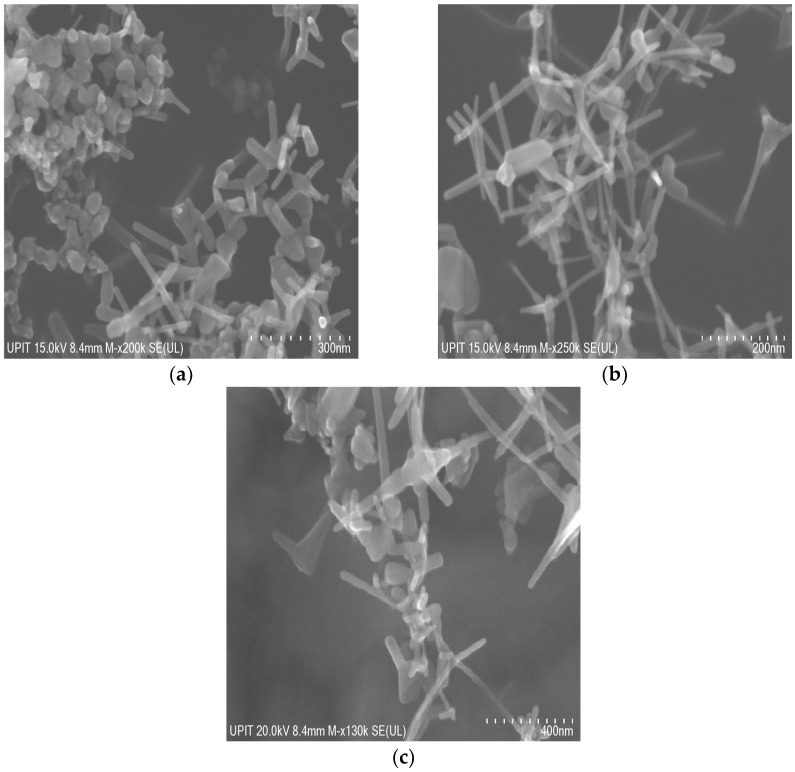
SEM micrographs of ZnO nanoparticles by SA corresponding to (**a**) 60 mbar, (**b**) 100 mbar, (**c**) 180 mbar.

Figure 4 shows the SEM morphologies of nanoparticles. It is visibly observed that the synthesized ZnO nanoparticles by SA are polyhedral and tetrapod in shape. 

The specific shape of the tetrapod is characterized by a center and four long and thin extremities. The length of all extremities sides is unequal at the top of the nanoparticle. This specific unequal length is due to the arrangement of the hexagonal unit cells (as supported by Table 3).

The growth mechanism of pure and Ni-doped ZnO nanoparticles by solar ablation is a complex procedure that is still not fully understood. The process of nanoparticle formation is the volatility-induced growth mechanism due to the solid-gas-solid transaction. In this mechanism, the zinc and oxygen atoms that are ablated from the ZnO target material are vaporized and then condensed on a substrate. The condensation process is driven by the difference in vapor pressure between the zinc and oxygen atoms. The zinc atoms are more volatile than the oxygen atoms, so they tend to condense first, forming a layer on the substrate. The oxygen atoms then condense on top of the zinc, completing the growth of the ZnO nanostructure by a process of self-assembly. In the case of ZnO/NiO targets ablation, along with Zn and O, they are vaporized and accelerated towards the substrate, where some of the Ni ions will become embedded in the ZnO lattice.

In the case of pure ZnO nanoparticles by SA at 60 mbar, the average length of polyhedral particles is 69.57 nm, while the average length of extremities of tetrapods is 72.01 nm and a diameter of around 13.31 nm. The polyhedral particles obtained by SA at 100 mbar are characterized by an average length of 71.14 nm, and the average length of extremities of tetrapods is 123.08 nm, which corresponds to an average diameter a little more than 20.20 nm. The longest extremities, on average 242 nm, were obtained at 180 mbar. All sizes increase with the effect of pressure, from 60 mbar to 180 mbar, which agrees with the XRD data.

**Figure 5:**
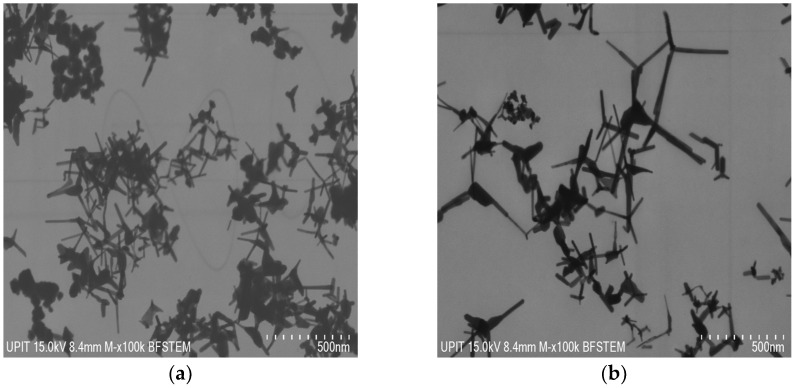

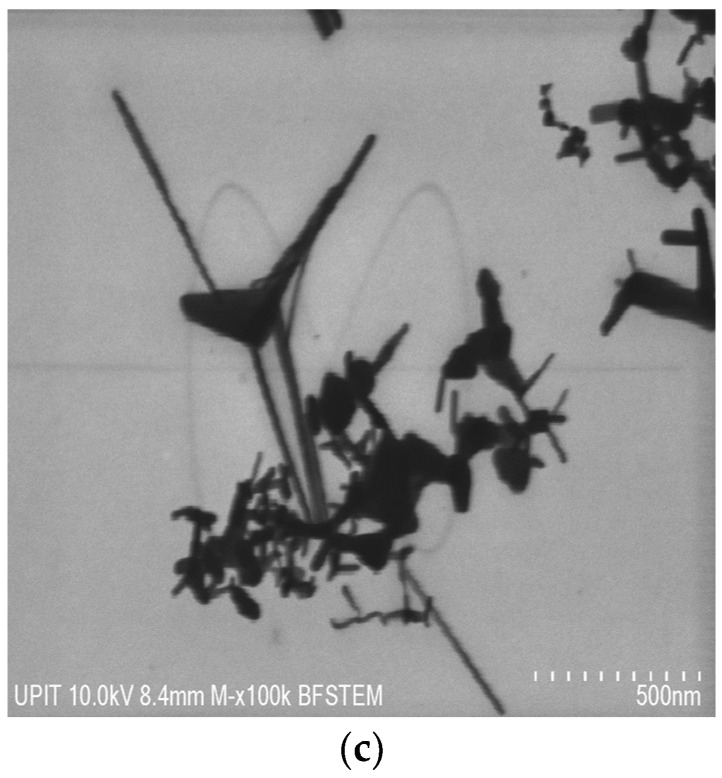
BF-STEM micrographs of ZnO nanoparticles by SA corresponding to (**a**) 60 mbar, (**b**) 100 mbar, (**c**) 180 mbar.

A detailed investigation of SANP/ZnO is shown in Figure 5 in the bright field mode in STEM at accelerating voltages of 15 kV and 10 kV.

The STEM images confirm the nanometric dimension and the two morphology types: polyhedral and tetrapods. 

To highlight whether there is symmetry in the distribution of tetrapods, the horizontal axis represents the size distribution of the length of the tetrapods. The vertical axis represents the number count or percentage of occurrences in the data for the particle size column.

The histograms of all SANP/ZnO samples show that a narrow range of size variation occurs.

**Figure 6:**
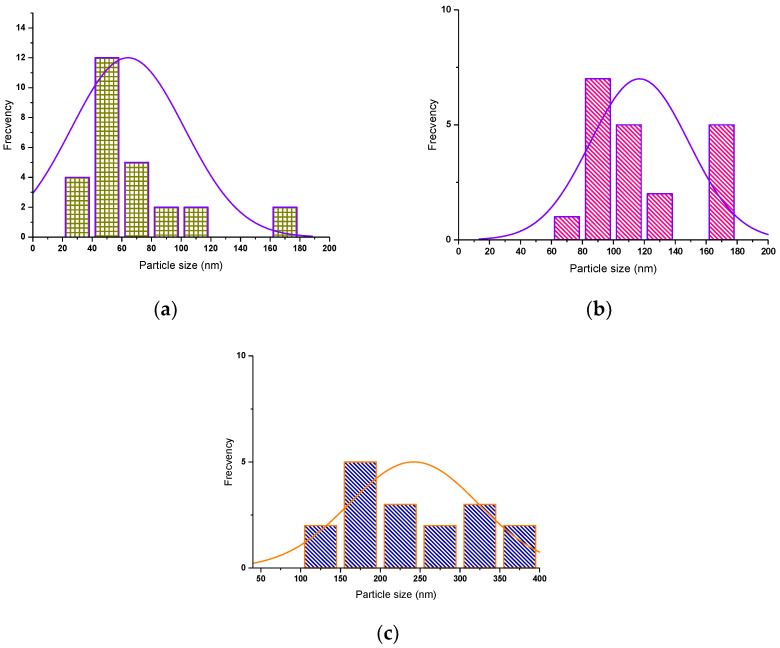
Tetrapod size histograms of SNP/ZnO samples at (**a**) 60 mbar; (**b**) 100 mbar; (**c**) 180 mbar.

The histograms of SANP/ZnO samples (Figure 6) present only one maximum peak corresponding to a monomodal distribution.

**Figure 7:**
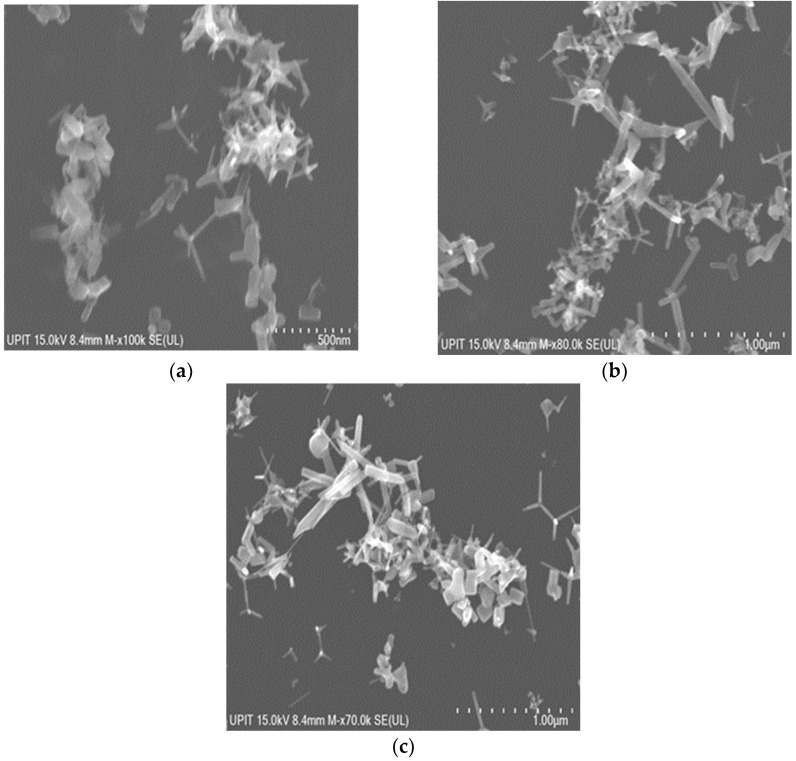
SEM micrographs of Ni-doped ZnO nanoparticles by SA corresponding to (**a**) 60 mbar, (**b**) 100 mbar, (**c**) 180 mbar.

The Ni-doped ZnO nanoparticles by SA also present a morphology composed of polyhedra and tetrapods whose average dimensions vary depending on the pressure in the solar reactor, as represented in Figure 7.

**Figure 8:**
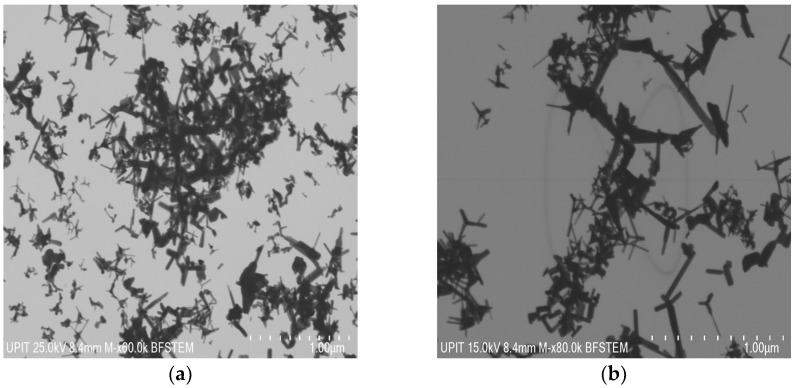

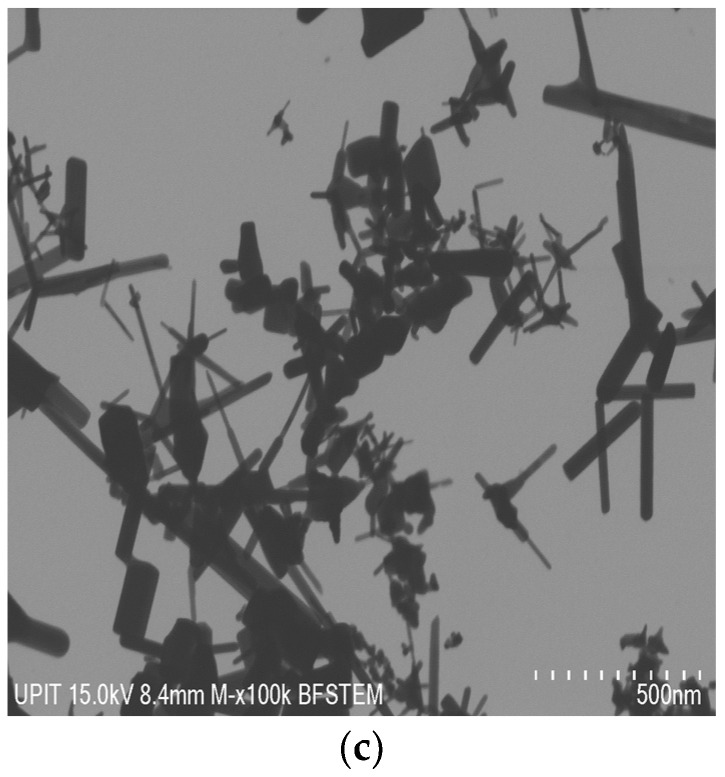
BF-STEM micrographs of Ni-doped ZnO nanoparticles by SA corresponding to (**a**) 60 mbar, (**b**) 100 mbar, (**c**) 180 mbar.

The internal microstructure can be visualized by BF-STEM, from Figure 8. 

The BF-STEM micrographs complete the remark that the morphologies are specific to solar ablation, namely tetrapods and polyhedra. 

The polyhedric particles range from 169.28 nm at 60 mbar to 128.9 nm at 100 mbar and 171.50 nm at 180 mbar. At 60 mbar, the extremities of tetrapodic nanoparticles are characterized by an average length of 145.45 nm and a diameter of 18.04 nm, while the pressure of 100 mbar helps to form longer whiskers with an average size of 128.9 nm and thicker (approximate diameter of 43.62 nm). All the results are strongly correlated with the size of the crystallites from XRD, which demonstrates their growth with the increase in pressure during the vapor-condensation mechanism.

The tetrapods with the longest extremities were obtained at 180 mbar, having an average length of 160.41 nm and a 52.15 nm in diameter).

**Figure 9:**
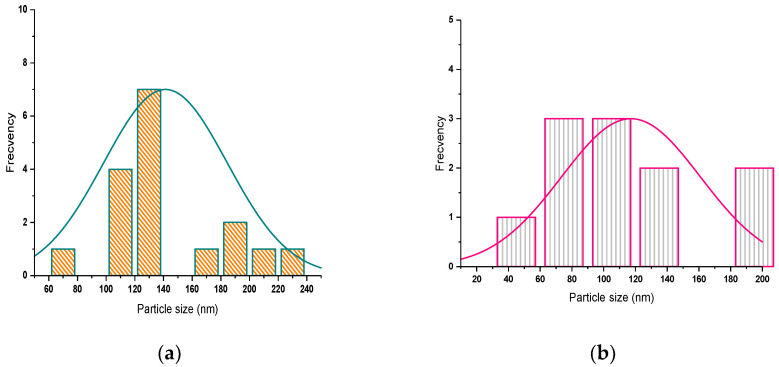

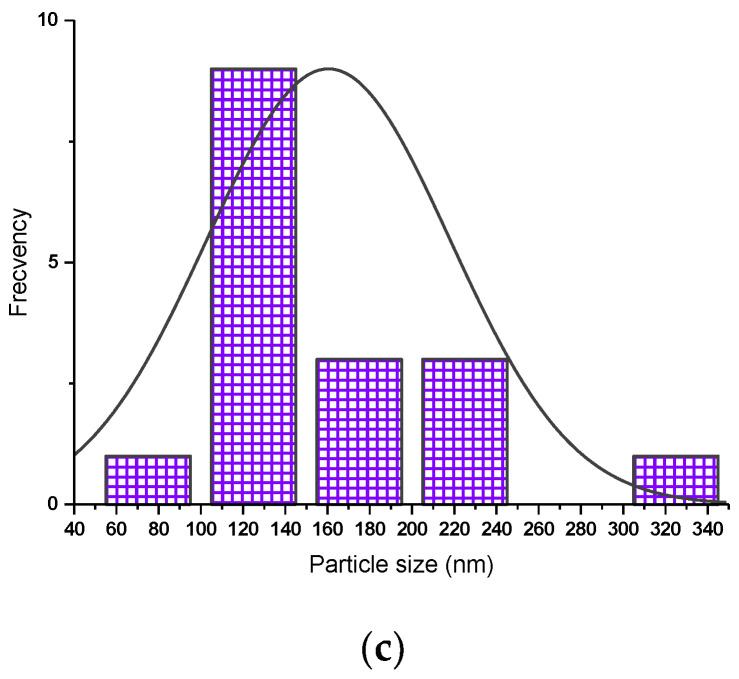
Tetrapod size histograms of SANP/NiZnO samples at (**a**) 60 mbar; (**b**) 100 mbar; (**c**) 180 mbar.

The size distribution of tetrapods is symmetrical, as can be seen from Figure 9.

Also, the frequency distribution of the length of whiskers of tetrapods has only one maximum, corresponding to a monomodal distribution. 

The tetrapod-like morphology, with uses in ammonia or hydrogen sensing [68], was also synthesized by the commercial ZnO heating process [69]. This type of arrangement was also obtained by thermal evaporation, favoring its use in the manufacture of solar cells to increase their energy efficiency [70]. The mentioned techniques and solar ablation have as a common factor the temperature and, as a difference, the energy that activates the evaporation process. 

Furthermore, the morphology of SA-prepared oxide nanocrystals strongly differs from those of samples obtained via sol-chemical reduction, the sol-gel method, hydrothermal method, or microemulsion, characterized by arrangement type wires [68,71], spherical morphology [72], or aggregates [73]. In addition, the SA sample quantities are smaller in comparison with those obtained via laser [3] or magnetron sputtering [4]. Our research provides an easy approach to show that the SA method leads to less agglomeration and better crystallinity.

### 3.3. Optical Characterization

**Figure 10:**
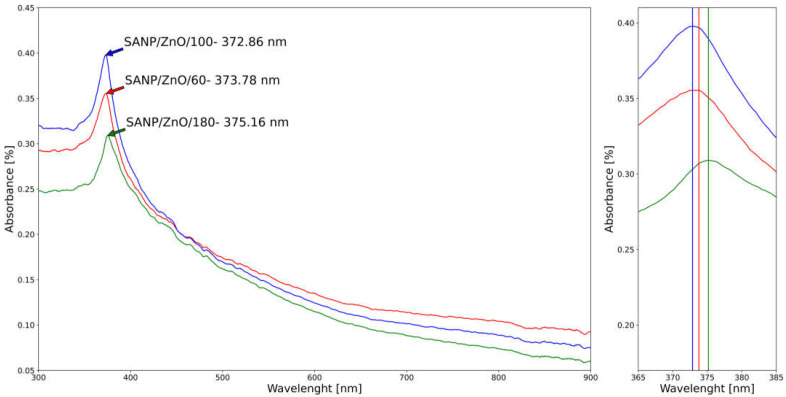
UV–VIS spectral region of ZnO nanoparticles obtained by SA: SANP/ZnO/60—red; SANP/ZnO/100—blue; SANP/ZnO/180—green; zoom of peaks.

**Figure 11:**
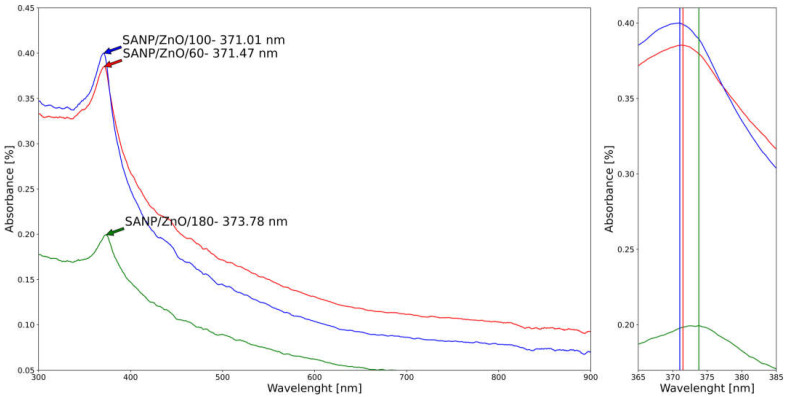
UV–VIS spectra region of Ni-doped ZnO nanoparticles obtained by SA: SANP/ZnNiO/60—red; SANP/ZnNiO/100—blue; SANP/ZnNiO/180—green; zoom of peaks.

Because the nanoparticles have unique optical properties that are sensitive to size and composition, the optical characteristics of pure and Ni-doped ZnO nanoparticles obtained by SA have been measured in the range of 200–1000 nm, as displayed in Figure 10 and Figure 11.

The maximum absorption peaks correspond to pure ZnO nanoparticles with small variations of wavelength of around 372–375 nm, influenced by the synthesis pressure. The UV-Vis spectra of Ni-doped ZnO are characterized by a blue shift of the absorption band in the UV region with a peak wavelength of around 371–373 nm. The increase in the band edge in addition to the blue shift of band edge for the Ni-doped samples by SA clearly indicates that Ni^2+^ ions are incorporated into the ZnO lattice [64]. The established reason for the blue shift in the band edge is due to interactions between the band electrons and the localized d-electrons of the Ni^2+^ ions [74]. The blue shift of the absorption band in Ni-doped ZnO can be used to tune the color of ZnO-based devices such as solar cells and LEDs. The blue shift is specific to the SA nanoparticle generator, unlike the hydrolytic method, in which is observed a red shift of the Ni-doped ZnO microspheres [72].

In addition, the red shift of SANP/ZnO compared to SANP/NiZnO corresponds to the materialization of clusters in the samples. Furthermore, in accordance with Deka and Joy, the displacement of the absorption band of SANP/NiZnO/180 towards lower wavelength as well as higher energy can be linked with the increase of the size of nanoparticles at the same pressure (33.02 average crystallite size of SANP/NiZnO/180 versus 31.03 nm average crystallite size of SANP/ZnO). 

## 4. Conclusions

The problem of controlling the size and shape of nanoparticles is a rather unexplored field. In fact, numerous processes are utilized for the synthesis of nanostructured materials and, in particular, for nanoparticle production. The solar energy has the potential to become an energy-generating source necessary for the elaboration of nanoparticles coming from commercially synthesized particles.

The elaboration of pure and Ni-doped ZnO particles by solar ablation fits into the characteristics of the nanometric field governed by domains, shapes, and unexpected properties. X-ray diffraction was the primary tool for probing nanostructure of samples. The average crystallite size varies from 24.23 nm to 33.02 nm. The characteristics of the ZnO lattice were influenced by the solar flux and pressure in the reactor. The Ni-doping was confirmed by XRD and UV-Vis patterns. The distribution histogram shows that a narrow range of size variation exists in each sample prepared by solar ablation. The optical properties reveal that the absorption band depends on the crystallite size and the doping element.

In addition, this result showed that solar ablation can also be used as an alternative to control and reduce particle dimensions, which is a strategy to improve the physical properties of nanomaterials.

Thus, from the excellent structural and optical properties of the samples, we expect that nickel-doped ZnO nanoparticles could be an efficient material for future exploration of CO_2_ and H_2_S gas-sensing performance ZnO sensors or LEDs. 

## Figures and Tables

**Table 1:** Comparison of the most used nanoparticle synthesis methods.

Method	Common Parameters	Specific Parameters	Advantages
Chemical reduction	**Solvent:** The solvent used in the reaction can affect the process reaction, the size of the nanoparticles produced, and the stability of the nanoparticles **Precursor:** The type of precursor used can affect the size, shape, and uniformity of the nanoparticles produced. **pH:** A lower pH will make the metal ions more stable and the reducing agent less stable, resulting in smaller nanoparticles. **Temperature:** Generally, a higher temperature will result in a faster reaction and smaller nanoparticles. However, too high of a temperature can also lead to the formation of defects. **Time:** A longer reaction time will result in a more complete reaction, but it can also lead to the formation of defects in the final product. **Stabilizer:** Common stabilizers include polymers, surfactants, and capping agents.	**Reducing agent concentration:** A higher concentration of reducing agent will result in a faster reaction and smaller nanoparticles. However, too high of a concentration can also lead to the formation of impurities. **Rate of reductant addition:** A slower rate of addition will result in a more uniform distribution of the reducing agent and smaller nanoparticles.	Advantages: Simple and versatile. Disadvantages: Can be difficult to control the size and shape of the nanoparticles.
Sol-gel synthesis		**Molar ratio:** A higher molar ratio will result in a higher concentration of the precursor in the solution, which can lead to the formation of larger particles.	Advantages: Can produce high-quality nanoparticles with controllable size and shape. Disadvantages: The process can be time-consuming and requires specialized equipment.
Hydrothermal synthesis		**Pressure:** A higher pressure will result in a higher solubility of the reactants and a faster reaction. **The temperature of the reaction** is typically in the range of 100–300 °C. **Reaction time:** The reaction time is in the range of 1–24 h.	Advantages: Synthesis of high-quality nanoparticles with controlled size and shape. Disadvantages: The hydrothermal process can be expensive and require specialized equipment.
Microemulsion synthesis		**Surfactant:** The type of surfactant used can affect the size, shape, and stability of the micelles, as well as the rate of nucleation and growth of the nanoparticles. **Cosurfactant:** The cosurfactant is typically a short-chain alcohol, such as ethanol or butanol, which can also affect the size, shape, and stability of the micelles. **Oil:** The type of oil used can affect the size, shape, and stability of the micelles, as well as the rate of nucleation and growth of the nanoparticles. **The temperature of the reaction** is in the range of 25–80 °C. **The reaction time** is in the range of 1−24 h.	Advantages: Produces high-quality nanoparticles with controllable size and shape. Disadvantages: Complexity and time-consuming. The need for specialized equipment.
Laser ablation	**Wavelength:** A wavelength that is well-absorbed by the material will result in more efficient ablation **Power:** A higher laser power will result in a deeper and more efficient ablation. **Duration:** The duration determines the amount of time that the beam is focused on the material being ablated and depends on the sublimation temperatures of the material. **Spot size:** A smaller spot size will result in a more localized and efficient ablation. **Atmosphere:** The atmosphere in which the ablation is performed can affect the ablation process. **Target material:** The properties of the target material, such as its reflectivity and absorption coefficient, can affect the ablation process.	**The laser wavelength** is typically in the range of 100–10,000 nm. **The laser power** is typically in the range of 1–100 watts. **Pulse repetition rate:** A higher pulse repetition rate will result in a faster ablation process. The pulse repetition of the laser rate is typically in the range of kHz to MHz.	Advantages: Afford the synthesis of nanoparticles with controlled size and shape. Disadvantages: The laser ablation process can be expensive and require specialized equipment.

**Table 2:** Elaboration parameters.

Sample	Solar Flux (W/m^2^)	Pressure (mbar)	Amount (g)
SANP/ZnO/60	974	60	0.5
SANP/ZnO/100	980	100	1.3
SANP/ZnO/180	910	180	0.4
SANP/NiZnO/60	953	60	0.8
SANP/NiZnO100	961	100	0.2
SANP/NiZnO/180	964	180	0.7

**Table 3:** Average crystallite size, lattice parameters, cell volume, and unit volume of SANP/ZnO samples.

Sample	D (nm)	a (Å)	c (Å)	c/a	V (Å^3^)	u (Å)
SANP/ZnO/60	24.23	3.252	5.211	1.6021	47.726	3.6273
SANP/ZnO/100	27.38	3.2531	5.212	1.6024	47.766	3.6283
SANP/ZnO/180	31.03	3.252	5.210	1.6021	47.712	3.6269

**Table 4:** Average crystallite size, lattice parameters, cell volume, and unit volume of SANP/NiZnO samples.

Sample	D (nm)	a (Å)	c (Å)	c/a	V (Å^3^)	u (Å)
SANP/NiZnO/60	27.88	3.252	5.208	1.60128	47.717	3.6270
SANP/NiZnO100	27.62	3.252	5.209	1.60124	47.723	3.6272
SANP/NiZnO/180	33.02	3.252	5.209	1.6014	47.725	3.6273

## Data Availability

Not applicable.
